# Geniposide activates the transcription of Ndufs8 and enhances PD-L1 blockade for inhibiting growth of osteosarcoma

**DOI:** 10.1016/j.jtcme.2025.04.002

**Published:** 2025-04-05

**Authors:** Fanghao Zheng, Tingting Zhao, Zhenyang Liu, Na Shao

**Affiliations:** a The Eighth School of Clinical Medicine, Guangzhou University of Chinese Medicine, Foshan, Guangdong, 528000, PR China; b Foshan Hospital of Traditional Chinese Medicine, Foshan, Guangdong, 528000, PR China; c Jinyu Laboratory College, Guangzhou Medical University, Guangzhou, Guangdong, 510000, PR China

**Keywords:** Osteosarcoma, Geniposide, Oxidative phosphorylation, Ndufs8, OVOL3

## Abstract

Geniposide, a key bioactive component of Gardenia jasminoides, is an iridoid glycoside known for its therapeutic potential in various diseases characterized by oxidative stress or inflammation, including atherosclerosis, brain disorders, and diabetes. However, its effects on osteosarcoma and the underlying mechanisms are unknown. Therefore, we explored the impact of geniposide on osteosarcoma growth through proteomic analysis. After subcutaneous tumorigenesis of osteosarcoma cells proteomic alterations were analyzed. GO and KEGG pathway analyses were performed to identify enriched pathways and critical proteins. Immunohistochemistry and Western blotting were conducted to measure the protein expression of AMPK, SIRT1, OVOL3, and Ndufs8. Flow cytometry, CCK-8, and colony formation assays were employed to assess osteosarcoma growth. ELISA assay was utilized to measure alterations in pyruvate, lactate, and ATP following geniposide treatment. ChIP were performed to confirm OVOL3 transcriptionally regulates Ndfus8. The results demonstrated that geniposide inhibited osteosarcoma growth by 45–65 % in tumor weight, with proteomic data revealing oxidative phosphorylation as the critical pathway. Geniposide reduced Ki-67 and PCNA expression by 30–55 %, while increasing apoptosis rates by 2.5-fold. Mechanistically, Ndufs8 was upregulated 1.8–2.3-fold by geniposide, enhancing ROS levels by 40–60 % and suppressing osteosarcoma cell viability by 50–70 %. Additionally, geniposide suppressed PD-L1 expression by 40–50 % and synergized with PD-L1 blockade. These findings reveal that geniposide inhibits osteosarcoma through dual mechanisms: Ndufs8-mediated oxidative phosphorylation activation and PD-L1/PD-L1 axis suppression, offering a novel therapeutic strategy. These findings suggest that geniposide is a promising therapeutic agent for osteosarcoma and may enhance the efficacy of immunotherapy combined with PD-L1 blockade.

## Graphical abstract

**Figure:**
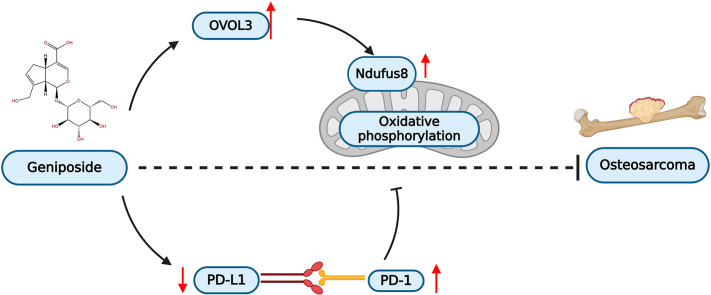

## 1 Introduction

Osteosarcoma is a primary malignant bone tumor that predominantly affects children, adolescents, and young adults.^1^^,^^2^ Despite significant advancements in medical technology over the past three decades, the 5-year survival rate for patients remains approximately 20 %.^3^^,^^4^ While substantial progress has been made in understanding the mechanisms and treatments for other types of tumors, research on osteosarcoma has been relatively limited. Therefore, it is cruical to further investigate the underlying mechanisms of osteosarcoma and develop targeted therapeutic strategies.

Geniposide, a key bioactive component of *Gardenia jasminoides*,^5^ is an iridoid glycoside, known for its therapeutic properties in treating various diseases associated with oxidative stress and inflammation, such as atherosclerosis, brain disorders, and diabetes.^5^, ^6^, ^7^, ^8^ Recent studies have also demonstrated its significant role in inhibiting tumor growth. *In vivo* experiments have shown that geniposide can mitigate aflatoxin B1-induced hepatocellular carcinoma by inhibiting γ-glutamyl transpeptidase activity.^9^ Other research indicates that geniposide suppresses proliferation, invasion, metastasis and angiogenesis both *in vitro* and *in vivo*.^10^ However, the precise mechanisms and molecular targets underlying its anti-cancer effects remain unclear.

A hallmark feature of tumors is the shift in cellular energy metabolism, where the primary energy source transitions from oxidative phosphorylation to glycolysis.^11^^,^^12^ However, most tumor mitochondria retain their ability to perform oxidative phosphorylation,^11^^,^^13^ and inhibition of this process is commonly observed in osteosarcoma.^14^, ^15^, ^16^ Thus, strategies aimed at reversing the inhibition of oxidative phosphorylation may offer potential therapeutic approaches for overcoming the challenges of osteosarcoma.

NADH: Ubiquinone Oxidoreductase Core Subunit S8 (Ndufs8) is a key enzyme in the mitochondrial respiratory chain responsible for NADH oxidation, ubiquinone reduction, and proton ejection from mitochondria.^17^ Located exclusively in the mitochondria, Ndufs8 plays a crucial role in oxidative phosphorylation, providing energy for cellular processes. Aberrant energy metabolism is one of the most prominent characteristics of tumors.^18^ Ovo Like Zinc Finger 3 (OVOL3) acts as a transcriptional that promotes DNA-binding transcription factor activity.^19^ Research by Chen JS has highlighted the significance of OVOL proteins in breast cancer expression and prognosis.^20^ But the role of OVOL3 in cancer remains poorly understood.

In this study, we investigated the effects of various concentrations of geniposide on the growth of osteosarcoma. The results showed that geniposide inhibited osteosarcoma growth. Proteomic analysis revealed that oxidative phosphorylation might be a critical pathway through which geniposide exerts its effects. Furthermore, we found that Ndufs8 activation, through the promotion of oxidative phosphorylation, contributed to the reversal of osteosarcoma growth. Geniposide enhanced Ndufs8 transcription by upregulating OVOL3 expression in osteosarcoma. Finally, *in vivo* experiments showed that geniposide reduced PD-L1 expression in osteosarcoma cells and enhanced the efficacy of PD-L1 blockade. These findings suggest that geniposide is a potential and novel agent for the treatment of osteosarcoma and could potentially enhance the effectiveness of immunotherapy when combined with PD-L1 blockade.

This work introduces groundbreaking insights into osteosarcoma therapeutics by uncovering the dual regulatory mechanisms of geniposide. First, we are the first to demonstrate that geniposide exerts a dual anticancer effect in osteosarcoma, simultaneously activating mitochondrial oxidative phosphorylation and enhancing PD-L1 blockade efficacy. Specifically, we reveal that geniposide uniquely promotes Ndufs8 transcription—a critical driver of mitochondrial respiration—through the upregulation of the transcription factor OVOL3, thereby reversing the suppressed oxidative phosphorylation characteristic of osteosarcoma. Second, our study pioneers the discovery of OVOL3 and Ndufs8 as novel functional regulators of metabolic reprogramming in osteosarcoma, establishing their roles in bridging transcriptional regulation with mitochondrial energy metabolism. Third, we identify for the first time that geniposide-mediated metabolic activation synergizes with immune checkpoint therapy by downregulating PD-L1 expression, creating a dual-targeted strategy that disrupts tumor growth while sensitizing osteosarcoma to PD-L1 blockade. These findings redefine geniposide as a multi-modal therapeutic agent and propose a paradigm shift in osteosarcoma treatment by integrating metabolic restoration with immune modulation.

## 2 Materials and methods

### 2.1 Cell lines and culture

Human osteosarcoma cells (143B) and mouse osteosarcoma cells (C3H) were purchased from ATCC. 143B cells were culturted in MEM medium (11095080, Gibco, USA), while C3H cells were cultured in DMEM medium (11885084, Gibco, USA), both supplemented with 10 % FBS (A5670701, Gibco, USA)and 1 % Penicillin-Streptomycin (HY-K1006, MedChemExpress, USA). All cells were maintained in an incubator at 37 °C with 5 % CO2 in a humidified atmosphere. Cells were acclimatized for 24 h prior to treatment. Both 143B and C3H cells were cultured until reaching passages 5–6, at which point they exhibited robust proliferation and were subsequently used in experimental procedures. Mycoplasma contamination was tested using BM-Cyclin (HY-K1059, MedChemExpress, USA) at the starting and end of the study, all cells were confirmed to be mycoplasma-free.

### 2.2 Cell transfection

Cells were transfected either with pcDNA4-Ndufs8 plasmid or si-OVOL3 at 50–60 % confluence. Transfections were performed using Lipofectamine 3000 (L3000015, Invitrogen, USA) according to the manufacturer's instructions. In brief, cells were transfected with 50 nM siRNA or 1 μg plasmid in 6 cm culture disk. After transfection, cells were incubated at 37 °C for 48 h. Following this incubation, cells were harvested, and used for subsequent experiments. The sequence of si-OVOL3 (143B) was 5′- UGUUGUGCCGGUGGCCCCCCC-3′, si-OVOL3 (C3H) sequence was 5′- UCGUGAAAGCCUUUACCACAA-3′ and the negative control (NC) sequence was 5′-UUCUCCGAACGUGUCACGU-3’.

### 2.3 Western blotting

Total Cellular proteins were extracted using the Whole Cell Lysis Assay (KGP2100, KeyGen BioTECH, China). Protein concentration was quantified with BCA Protein Quantitation Assay (KGP902, KeyGen BioTECH, China). The processes of protein extraction and quantification were performed according to the manufacturer's instruction manuals. A total of 30 μg of protein was separated by SDS-PAGE (Bio-Rad, USA), and transferred electrophoretically to a polyvinylidene fluoride (PVDF) membrane. The PVDF membrane was then blocked with 3 % Bovine Serum Albumin (9048-46-8, Absin, China) for 1 h, and incubated with specific primary antibodies overnight at 4 °C according to the recommended dilution ratios. The primary antibodies used were anti-AMPK (1:2000, 10929-2-AP, Proteintech, China), anti-SIRT1 (1:1000, 8469S, CST, USA), anti-GAPDH (1:5000, 60004-1-lg, Proteintech, China). The corresponding second antibodies (BM2020, Boster, China) were incubated for 2 h at room temperature. Protein signals were detected using BeyoECL Plus (P0018S, Beyotime, China).

### 2.4 RNA extraction and reverse transcription-quantitative PCR (RT-qPCR)

Total RNA was extracted using RNAiso Plus (9108, Takara, Japan), and reverse transcription was performed using the PrimerScript RT Master Mix (RR036Q, Takara, Japan) according to the manufacturer's instructions. RT-qPCR was performed using TB Green Fast qPCR Mix (RR430S, Takara, Japan) on a Bio-Rad MyiQ iCycler qPCR instrument (Bio-Rad, USA). The 2^−ΔΔCt^ method was used to calculate the relative quantification of target gene expression, with GAPDH serving as the internal control. Primer sequences are provided in Supplemental Table 1.

### 2.5 Immunohistochemistry

Tissue samples were fixed with Paraformaldehyde (BL539A, Biosharp, China) after immersion in normal saline. Paraffin sections were dewaxed in xylene (1001–0220, Yongda Chemical, China) and rehydrated through a graded series of alcohols. Antigen retrieval was performed by incubating sections in citrate buffer (P4809-50TAB, Merck, Germany) at 90 °C for 30 min. After cooling to room temperature, the sections were transferred to Tris buffered saline (TBS; 0.15 M NaCl, 0.1 M Tris-Cl pH 7.4). Endogenous peroxidase activity was blocked by incubating sections in 0.1 % NaN_3_, and 1 % H_2_O_2_, in TBS for 15 min. All blocking and antibody binding incubations were performed in a humidified chamber. After a brief wash in TBS, the sections were blocked with 10 % non-immune donkey serum (36136ES60, Yeasen, China) for 30 min.

### 2.6 Colony formation assay

Cells were seeded in 3.5 cm culture dishes and cultured for 20 days with treatment. For the human osteosarcoma cell line 143B, 200 to 300 cells per culture dish were inoculated, and for the mouse osteosarcoma cells, 100 to 200 cells per dish. This cell density ensures an adequate number of colonies for subsequent analysis while preventing overcrowding, which could impact colony quality and morphology. Afterward, the cells were washed twice with PBS (C0221A, Beyotime, China), fixed with 4 % paraformaldehyde (BL539A, Biosharp, China) for 15 min at room temperature, and stained with 0.1 % crystal violet (B1087, Applygen Technologies, China) for 15 min. The cells were then washed twice. The cells were then washed twice with PBS, and colony numbers were counted to assess cell proliferation.

### 2.7 Cell viability assay

Cell viability was assessed using the CCK-8 assay (CK04, Dojindo, Japan). In brief, cells were seeded in 96-well plates at a density of 4000 cells per well in 100 μL DMEM with 10 % FBS for 24 h. After replacing the medium, cells were treated according to the experimental groups. Then, 10 μL of CCK-8 regent was added to each well, and the cells were incubated for 2 h. Absorbance at 450 nm was measured using a microplate reader (BioTek, USA). DMEM/FBS and CCK-8 without cells were used as blanks. Cell viability (%) = (A450_treatment_- A450_blank_)- (A450_control_- A450_blank_).

### 2.8 Cell apoptosis detection

Cell apoptosis was evaluated by flow cytometry (BD Biosciences, USA). Cells (1 × 10^6^) were digested with Trypsin Solution without EDTA (C0205, Beyotime, China), and washed twice with PBS. The cells were resuspended in 500 μL of 1 × binding buffer (BL1890A, Biosharp, China), then 2 μL of Annexin V-FITC (C1062S, Beyotime, China) and 2 μL of Propidium Iodide (ST511, Beyotime, China) were added. After incubation in the dark for 10 min, cell apoptosis was detected by flow cytometry.

### 2.9 Cell ROS detection

The intracellular reactive oxygen species (ROS) levels were measured by flow cytometry. After treatment and washing twice with PBS, cells were resuspended in PBS and stained with 10 μM H2DCFDA (4091-99-0, MedChemExpress, USA) for 30 min in the dark. ROS levels were assessed by flow cytometry.

### 2.10 Proteomics analysis and bioinformatics analysis

Proteomic analysis was performed on tissue samples from the Model group, Low dosage group, and High dosage group, outsourced to Shanghai Zhongke New Life Biotechnology Co., Ltd. Differentially expressed proteins were based on the following criteria: *p* < 0.05 and fold change (FC) > 1.2 or < 0.83. Proteins were compared between the Low dosage/High dosage groups and the Model group. Gene Ontology (GO) annotation and Kyoto Encyclopedia of Genes and Genomes (KEGG) pathway analysis were conducted using the Database for Annotation, Visualization and Integrated Discovery (DAVID) database^21^ (https://david.ncifcrf.gov/).

### 2.11 Metabolic measurements

The cell culture medium was collected and centrifuged at 400 g for 5 min. The concentrations of pyruvate, lactic acid, and ATP were measured according to the instructions. Pyruvate concentration was measured using the Pyruvate assay kit (A081-1-1, Nanjing Jiancheng Bioengineering Institute, China), lactic acid concentration using the Lactic Acid Assay kit (A019-2-1, Nanjing Jiancheng Bioengineering Institute, China), and ATP concentration using the ATP assay kit (S0026, Beyotime, China).

### 2.12 *In vivo* experiments

Four-week-old male BALB/c or BALB/c Nude mice were maintained on a normal chow diet in specific pathogen free conditions, in accordance with guidelines approved by the Institutional Animal Care and Use Committee of Southern Medical University (Ethical approval number: L2020112). 1.5 × 10^7^ cells were subcutaneously injected into the right flank of the mice (n = 4 per experimental group). Tumor volumes were measured every 4 days using calipers and calculated using formula (L × W^2^)/2, where L is the longest diameter and W is the shortest diameter. Tumor growth was monitored weekly. Anti-PD-L1 (RMP1-14; BioXcell), 200 μg for per mouse was administered twice a week. After 42 days of treatment, the mice were euthanized, and tumor weights were measured. Tumors were then resected for histological and proteomic analysis.

### 2.13 Bioinformatics analysis

Principal Component Analysis (PCA) was performed using ClustVis^22^ (https://biit.cs.ut.ee/clustvis/). Differential proteins were screened based on fold change >1.2 or <0.83, and *p* < 0.05. A Venn diagram was generated using the Bioinformatics & Evolutionary Genomics (http://bioinformatics.psb.ugent.be/webtools/Venn/). KEGG pathway and GO analysis were conducted using the DAVID database^21^ (https://david-d.ncifcrf.gov/).

### 2.14 Statistical analysis

Statistical analysis was performed using IBM SPSS Statistics 21.0 software (IBM, USA). Data are presented as mean ± standard deviation (SD) from three independent experiments. Differences between groups were analyzed using an unpaired Student's t-test, and one-way ANOVA was used for comparisons among three groups. *P* < 0.05 was considered statistically significant.

## 3 Results

### 3.1 Geniposide inhibited *in vivo* growth of osteosarcoma in tumor-bearing nude mice

**Fig. 1:**
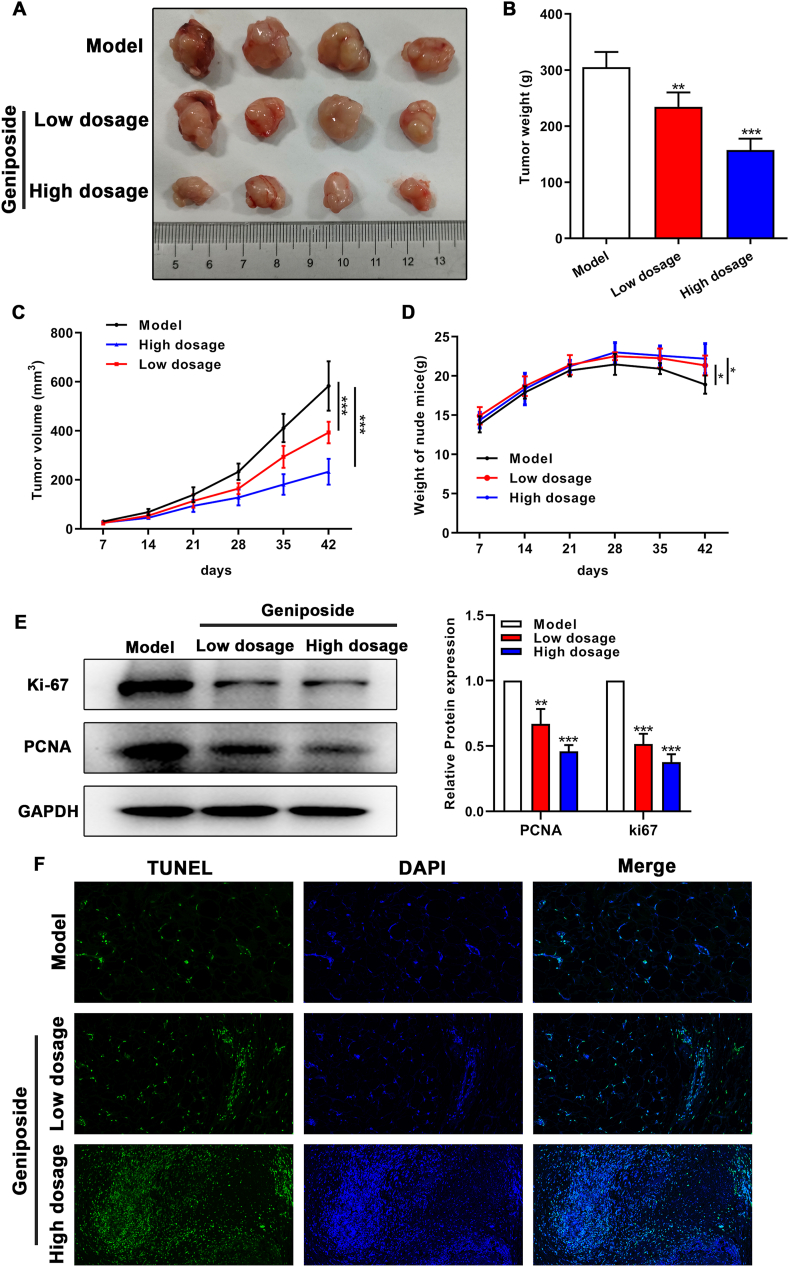
Geniposide inhibited *in vivo* growth of osteosarcoma in tumor-bearing nude mice. (A) Representative images of the subcutaneous tumors at the end of the experiment. (B) Weight of the subcutaneous tumors were analyzed. (C) Subcutaneous tumor growth curves were constructed by plotting tumor volumes against time. (D) Weight of nude mice growth curves were constructed by plotting weight against time. (E) Representative protein expression of Ki-67 and PCNA in subcutaneous tumor tissues. (F) TUNEL staining of subcutaneous tumor tissues. ∗*p*<0.05, ∗∗*p*<0.01, ∗∗∗*p*<0.001.

To investigate the tumor-suppressive effects of geniposide on osteosarcoma, nude mice with tumor-bearing were intraperitoneally injected with varying concentrations of geniposide. As shown in Fig. 1A, geniposide inhibited osteosarcoma growth in a dose-dependent manner. Tumor weights were significantly lower in the geniposide-treated groups compared to the model group, and tumor weight decreased with increasing concentrations of geniposide (Fig. 1B). Tumor size was measured weekly to assess the temporal effects of geniposide on osteosarcoma growth. Although no significant inhibitory effects were observed during the early stages (Day 7–21), geniposide significantly suppressed osteosarcoma growth over time (Fig. 1C). Body weight data indicated that mice in the geniposide treatment gained more weight compared to the model group (Fig. 1D). To assess cell proliferation, protein levels of Ki-67 and PCNA were measured in subcutaneous tumors. geniposide treatment reduced the expression of Ki-67 and PCNA in a dose-dependent manner (Fig. 1E). Additionally, TUNEL assay results revealed an increase in EdU-positive cells with higher concentrations of geniposide, indicating enhanced cell apoptosis (Fig. 1F). These *in vivo* findings demonstrate that geniposide effectively inhibits the growth of osteosarcoma in the tumor-bearing nude mice.

### 3.2 Bioinformatics analysis of subcutaneous tumors treated with geniposide

**Fig. 2:**
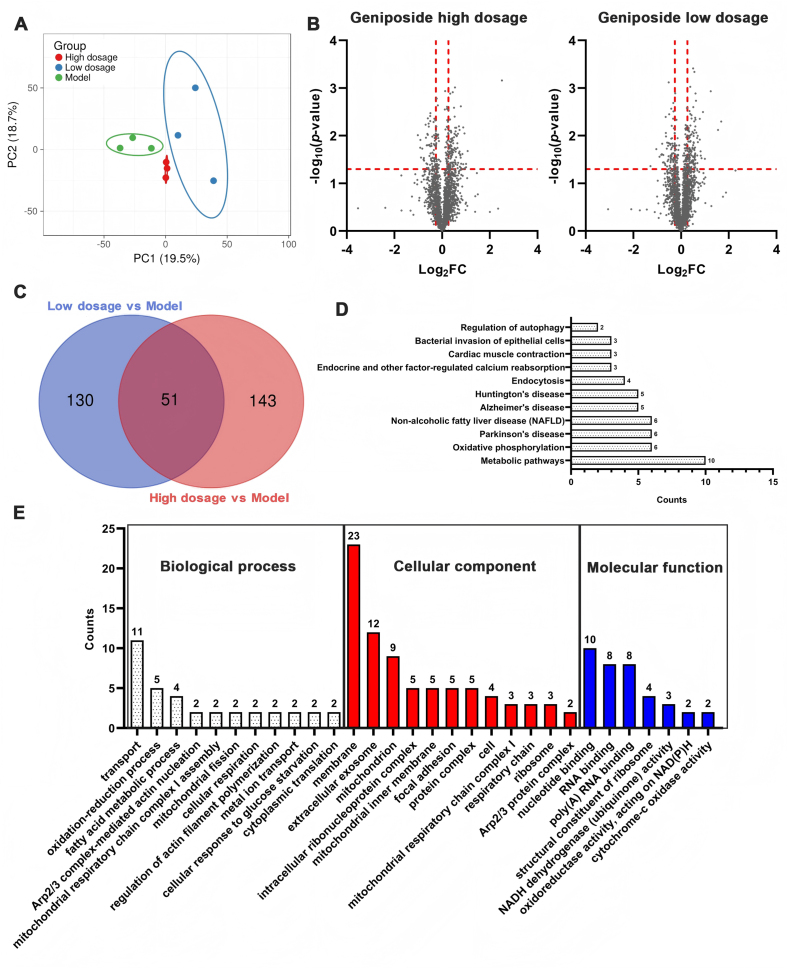
Bioinformatics analysis of subcutaneous tumors treated with Geniposide. (A) PCA analysis was performed to the effect of Geniposide on protein expression in Proteomics. (B) Volcano plots were performed to analyse the significant proteins (Fold change >1.2 or < 0.83, and p<0.05, Low dosage group vs Model group, and High dosage group vs Model group). (C) Venn diagram was used to analysis the common significant proteins. (D) KEGG pathway analysis was performed to identify candidate pathways Geniposide involved. (E) GO analysis was performed to identify candidate pathways Geniposide involved.

Proteomics analysis was performed on tumor tissues before and after geniposide treatment to explore its inhibitory effects on osteosarcoma. Detailed date are provided in Supplemental Table 2. PCA was conducted on the proteomic data, showing that geniposide treatment, as well as its varying concentrations, affected overall protein expression (Fig. 2A). These results confirm successful model construction and validate the quality of the proteomics data for subsequent analysis. Volcano plot analysis, with thresholds set at a fold change >1.2 or <0.83, and *p* < 0.05, revealed 182 significant proteins in the Low dosage group versus the Model group, and 195 significant proteins in the High dosage group versus the Model group (Fig. 2B). A Venn diagram identified 51 common significant proteins shared between the Low dosage/Model and High dosage/Model groups (Fig. 2C). KEGG pathway analysis of these common proteins indicated that geniposide affected metabolic process and oxidative phosphorylation in osteosarcoma (Fig. 2D). Detail results are provided in Supplemental Table 3. GO analysis further revealed alterations in processes such as transport and oxidation-reduction, as well as in components like the membrane and extracellular exosome, and molecular functions such as nucleotide binding and RNA binding (Fig. 2E). Detailed results are also available in Supplemental Table 3. These findings indicate that geniposide regulates a series of pathways, particularly those involving oxidative phosphorylation, to inhibit osteosarcoma growth.

### 3.3 Effects of geniposide on oxidative phosphorylation and osteosarcoma cell proliferation

**Fig. 3:**
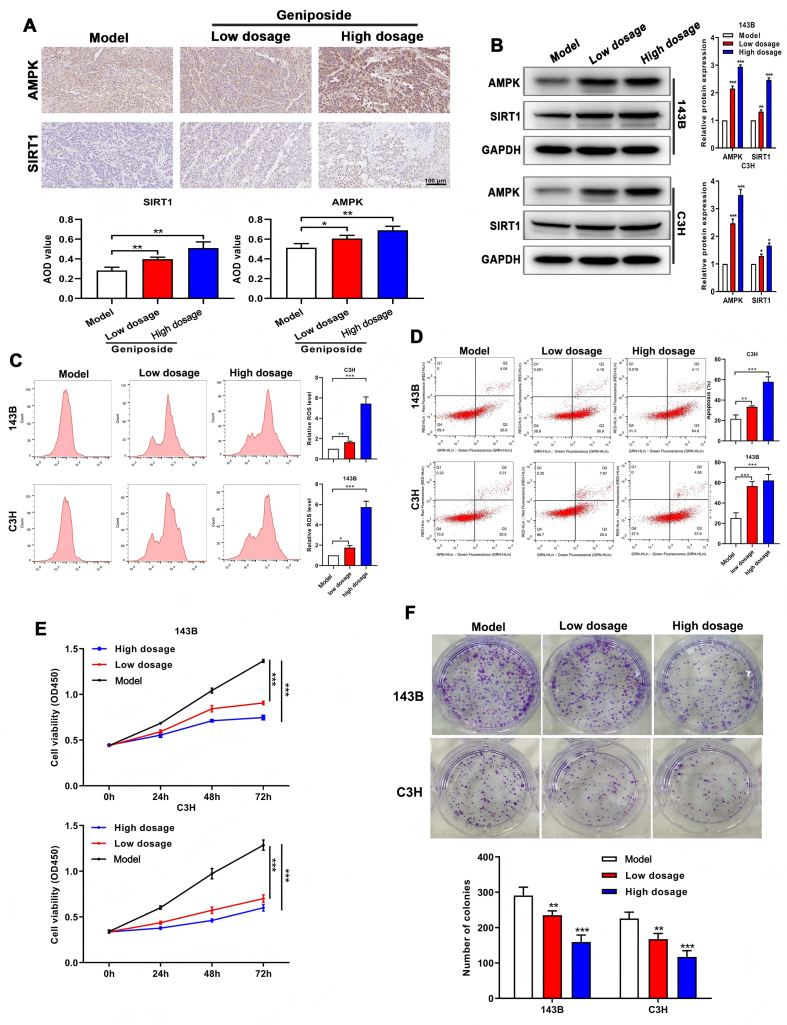
Effects of Geniposide on oxidative phosphorylation and osteosarcoma cell proliferation. (A) IHC from subcutaneous tumors reflected that Geniposide increased the expression of AMPK and SIRT1. (B) The protein expression of AMPK and SIRT1 were increased with Geniposide treatment in 143B and C3H cells. (C and D) The ROS levels and cell apoptosis rates were elevated with Geniposide treatment in 143B and C3H cells. (E) The cell viability was decreased with Geniposide treatment in 143B and C3H cells. (F) Colony formation assay reflected that the proliferation ability was decreased with Geniposide treatment in 143B and C3H cells. ∗*p*<0.05, ∗∗*p*<0.01, ∗∗∗*p*<0.001.

AMPK and SIRT1 are known indicators of oxidative phosphorylation activation. IHC results indicated that the expression of both AMPK and SIRT1 was upregulated following geniposide treatment, indicating the activation of oxidative phosphorylation in osteosarcoma (Fig. 3A). We further measured the protein levels of AMPK and SIRT1 in 143B and C3H cells, which demonstrated a dose-dependent increase in both proteins after geniposide treatment (Fig. 3B). Flow cytometry was used to asses ROS levels and apoptosis rates with geniposide treatment in 143B and C3H cells following geniposide treatment. geniposide increased ROS levels and significantly elevated apoptosis rates in both cell lines (Fig. 3C and D). CCK-8 assays revealed a decrease in cell viability with increasing geniposide concentration (Fig. 3E), consistent with the apoptosis data. To further evaluate cell proliferation, colony formation assays were performed, and geniposide treatment resulted in decreased cell proliferation ability in both 143B and C3H cells (Fig. 3F). These results collectively suggest that geniposide activates oxidative phosphorylation and inhibits osteosarcoma cell proliferation.

### 3.4 Geniposide altered the glycolytic phenotype in osteosarcoma cells, but not healthy cells

**Fig. 4:**
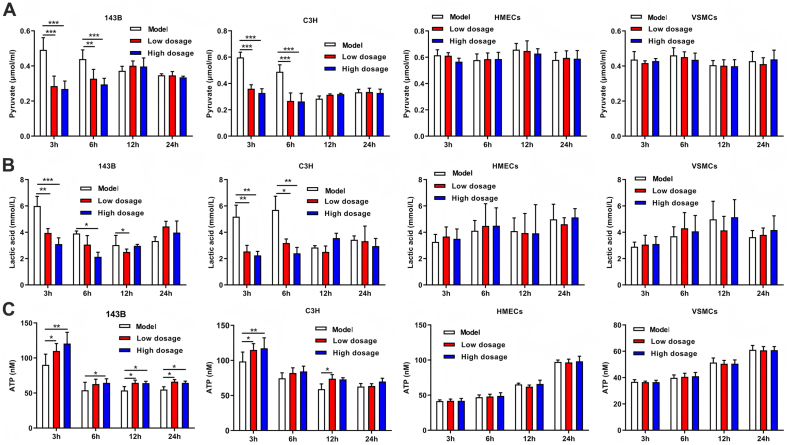
Geniposide altered the glycolytic phenotype in osteosarcoma cells, but not healthy cells. (A) The concentration of pyruvate in the culture media of osteosarcoma cells (143B and C3H), and healthy cells (HMECs and VSMCs cells). (B) The concentration of lactic acid in the culture media of osteosarcoma cells (143B and C3H), and healthy cells HMECs and VSMCs cells. (C) The concentration of ATP in the culture media of osteosarcoma cells (143B and C3H), and healthy cells (HMECs and VSMCs cells). ∗*p*<0.05, ∗∗*p*<0.01, ∗∗∗*p*<0.001.

Pyruvate is produced from glucose through glycolysis,^23^ while lactic acid is an metabolic byproduct that contributes to tumor initiation and development.^24^ Both oxidative phosphorylation and anaerobic glycolysis produce ATP, but oxidative phosphorylation is far more efficient in ATP generation.^25^^,^^26^ Tumor cells often rely more on anaerobic glycolysis for ATP production due to the inhibition of oxidative phosphorylation.^27^, ^28^, ^29^ We measured the concentrations of pyruvate, lactic acid, and ATP in the cell culture supernatants at 3, 6, 12, and 24 h in 143B and C3H cells treated with geniposide. The results reflected geniposide treatment decreased pyruvate and lactic acid levels, while ATP levels were increased in 143B and C3H cells. However, geniposide did not alter the energy metabolism pathway in normal cells, including HMECs and VSMCs (Fig. 4). These findings suggest that geniposide alters the glycolytic phenotype specifically in osteosarcoma cells, without affecting healthy cells.

### 3.5 Effects of Ndufs8 on oxidative phosphorylation and osteosarcoma cell proliferation

**Fig. 5:**
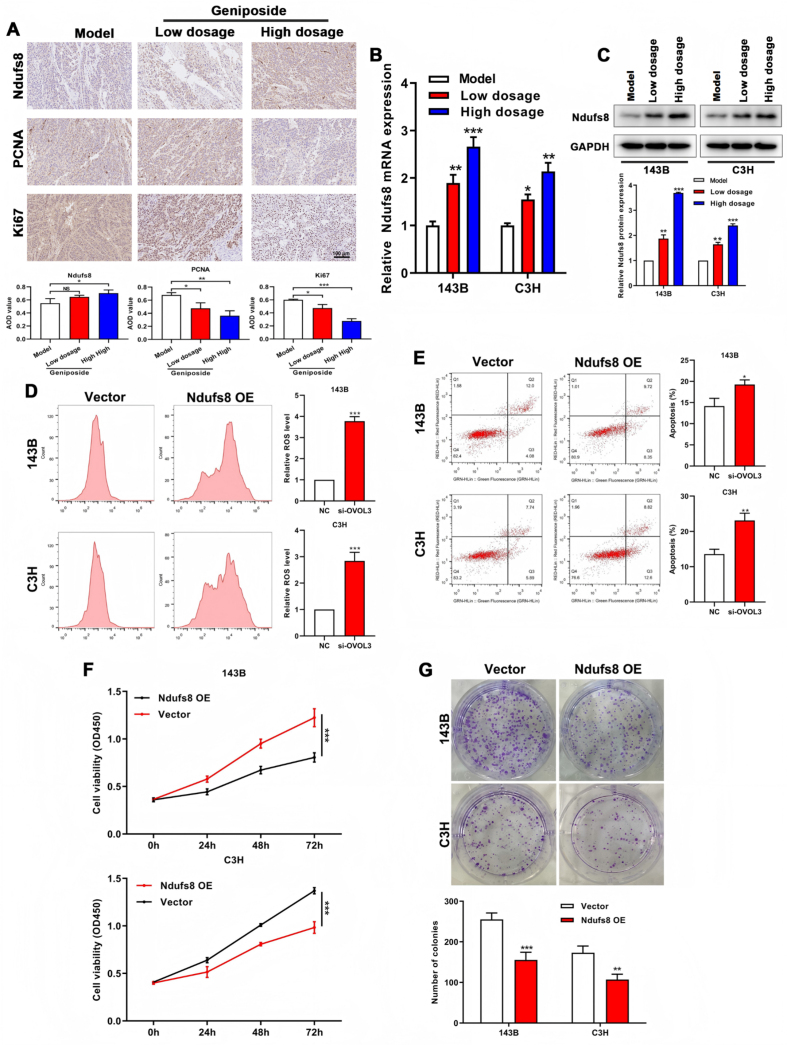
Effects of Ndufs8 on oxidative phosphorylation and osteosarcoma cell proliferation. (A) IHC from subcutaneous tumors reflected that Geniposide increased the Ndufs8 expression and decreased PCNA and Ki67 expression. (B and C) The mRNA and protein expression of Ndufs8 were increased with Geniposide treatment in 143B and C3H cells. (D and E) The ROS levels and cell apoptosis rates were elevated with Ndufs8 overexpression in 143B and C3H cells. (F) The cell viability was decreased with Ndufs8 overexpression in 143B and C3H cells. (G) Colony formation assay reflected that the proliferation ability was decreased with Ndufs8 overexpression in 143B and C3H cells. Vector: empty vector control for overexpression experiments; NC: scramble siRNA control for knockdown experiments. ∗p<0.05, ∗∗p<0.01, ∗∗∗p<0.001.

Proteomic analysis indicated that Ndufs8 is involved in oxidative phosphorylation. IHC analysis of osteosarcoma tissues revealed increased expression of Ndufs8, while the levels of PCNA and Ki67, markers of proliferation, were decreased upon geniposide treatment (Fig. 5A). Both mRNA and protein levels of Ndufs8 were upregulated in 143B and C3H cells following geniposide treatment, with expression increasing in a dose-dependent manner (Fig. 5B and C). We then overexpression Ndufs8 in 143B and C3H cells, and measured ROS levels and apoptosis rates (Fig. 5D and E), confirming that Ndufs8 suppresses osteosarcoma cell growth. Furthermore, cell viability assays demonstrated that Ndufs8 overexpression suppressed cell viability in both cell lines (Fig. 5F). Colony formation assays revealed that Ndufs8 overexpression decreased the proliferation ability of C3H and 143B cells (Fig. 5G). The results confirm that Ndufs8 activates oxidative phosphorylation and inhibits osteosarcoma cell proliferation.

### 3.6 Transcription factor OVOL3 promoted the transcriptional activity of Ndufs8

**Fig. 6:**
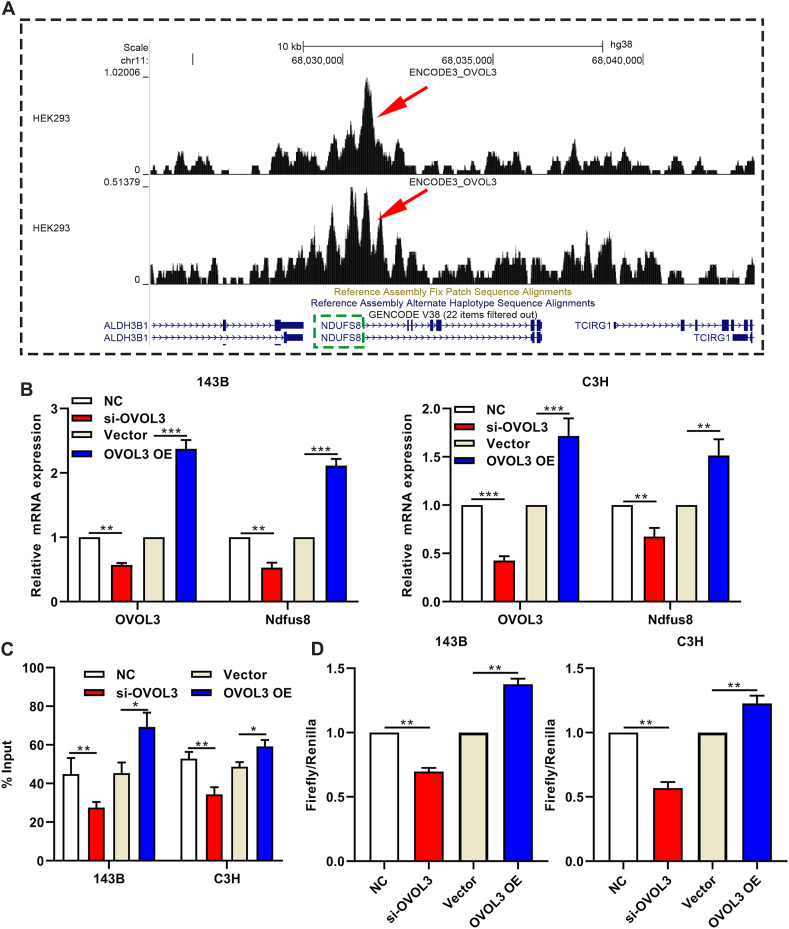
Transcription factor OVOL3 promoted the transcriptional activity of Ndufs8. (A) ChIP-seq analysis revealed that OVOL3 binds to the promoter region of Ndufs8. (B) The mRNA levels of OVOL3 and Ndufs8 in 143B and C3H cells with si-OVOL3 or OVOL3 overexpression plasmid transfection. (C) ChIP-qPCR was performed to identify the binding of OVOL3 on Ndufs8 promoter region. (D) Luciferase reporter gene was performed to identify the binding of OVOL3 on Ndufs8 promoter region. ∗p<0.05, ∗∗p<0.01, ∗∗∗p<0.001.

The expression of proteins is primarily regulated by their transcription and degradation. Based on data from the Cistrome database, we speculated that OVOL3 could bind to the promoter region of Ndufs8 to activate its transcription (Fig. 6A). ChIP-seq data from the Cistrome database indicated a binding signal at the Ndufs8 transcriptional start site (TSS) between −350bp and −400 bp. This suggests that OVOL3 binds to the Ndufs8 promoter region at this location. To further investigate, we transfected 143B and C3H cells with either si-OVOL3 and OVOL3 overexpression plasmid (Fig. 6B). mRNA levels of Ndufs8 were increased upon OVOL3 overexpression and decreased following si-OVOL3 transfection (Fig. 6B). ChIP-qPCR confirmed that OVOL3 binding to the Ndufs8 promoter region was enhanced after OVOL3 overexpression, while si-OVOL3 transfection reduced the binding (Fig. 6C). Dual-luciferase reporter assays further supported this conclusion, showing that OVOL3 promotes Ndufs8 transcriptional activity (Fig. 6D). These results indicate that OVOL3 binds to the Ndufs8 promoter and promotes its transcriptional activity.

### 3.7 Geniposide activated the transcription of Ndufs8 and inhibited the growth of osteosarcoma by increasing the expression of OVOL3

**Fig. 7:**
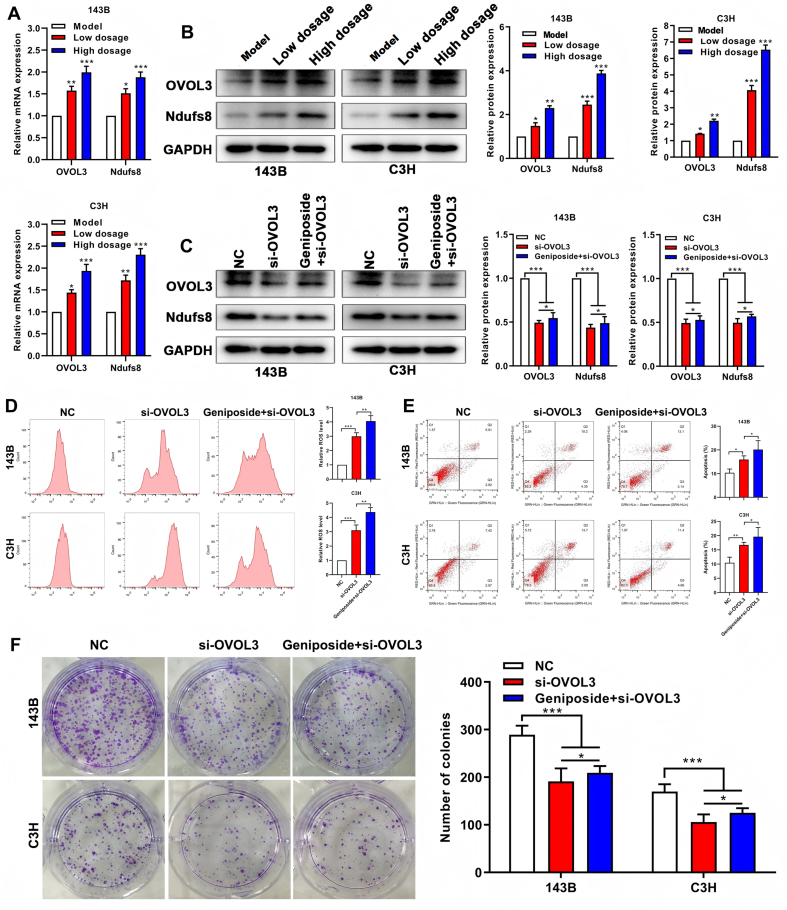
Geniposide activated the transcription of Ndufs8 and inhibited the growth of osteosarcoma by increasing the expression of OVOL3. (A and B) The mRNA and protein expressions of OVOL3 and Ndufs8 were increased as the concentration of Geniposide increases in 143B and C3H cells. (C) Geniposide reversed si-OVOL3 mediated Ndufs8 downregulation in 143B and C3H cells. (D and E) The ROS levels and cell apoptosis rates were detected in 143B and C3H cells with si-OVOL3 transfection and Geniposide treatment. (F) Colony formation assay reflected that the proliferation ability in 143B and C3H cells with si-OVOL3 transfection and Geniposide treatment. ∗p<0.05, ∗∗p<0.01, ∗∗∗p<0.001.

Having established the individual effects of geniposide and Ndufs8 on osteosarcoma, we explored whether geniposide activates Ndufs8 transcription via OVOL3. As showen in Fig. 7A and B, geniposide treatment increased both mRNA and protein expression of OVOL3 and Ndufs8 in 143B and C3H cells in a dose-dependent manner. Moreover, the protein expression of Ndufs8 was elevated in si-OVOL3 transfected 143B and C3H cells treated with geniposide (Fig. 7C). Flow cytometry revealed increased ROS levels and higher apoptosis rates in si-OVOL3 transfected cells treated with geniposide (Fig. 7D and E). Additionally, the number of colonies increased in si-OVOL3 transfected cells treated with geniposide (Fig. 7F). These findings demonstrate that geniposide activates the transcription of Ndufs8 and inhibits the growth of osteosarcoma by increasing the expression of OVOL3.

### 3.8 Geniposide enhances PD-L1 blockade effect by downregulating PD-L1 expression in osteosarcoma

**Fig. 8:**
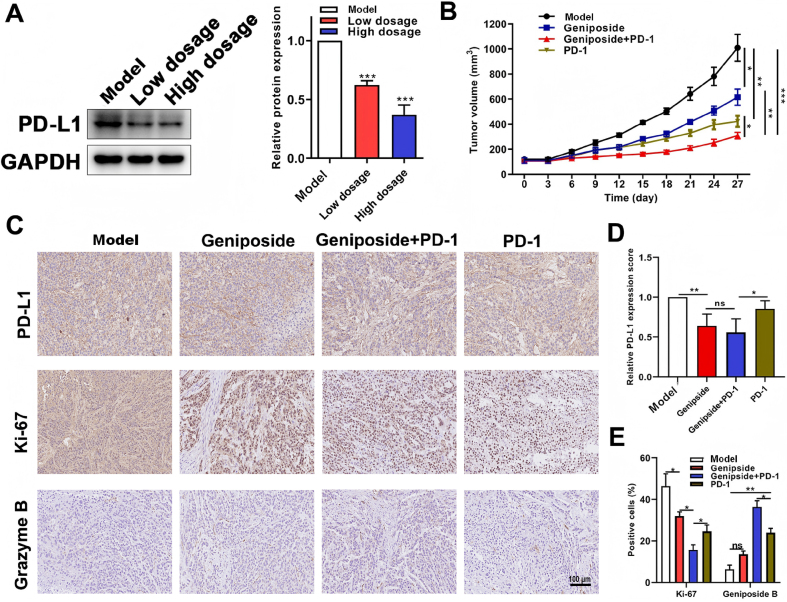
Geniposide enhances PD-L1 blockage effect by downregulating PD-L1 expression in osteosarcoma. (A) The protein expression of PD-L1 was reduced with Geniposide treatment in 143B cells. (B) C3H mice osteosarcoma cells were used to construct a xenograft model with BALB/C mice to evaluate the combination effect of Geniposide and PD-L1 blockage. Subcutaneous tumor growth curves were constructed by plotting tumor volumes against time. (C-E) IHC analysis of PD-L1, Ki67 levels and Grazyme B positive cells in steosarcoma xenografts with different treatment. ∗p<0.05, ∗∗p<0.01, ∗∗∗p<0.001.

Geniposide upregulated the expression of AMPK in osteosarcoma cells, and AMPK activation is known to regulate PD-L1 expression. Our results showed that treatment with geniposide reduced PD-L1 expression in osteosarcoma cells (Fig. 8A). We also evaluated the combined effect of geniposide and PD-L1 blockade *in vivo* (Fig. 8B)*.* Notably*,* geniposide enhanced the effect of PD-L1 blockade in the osteosarcoma cell xenograft mouse model. IHC analysis revealed that geniposide treatment decreased PD-L1 expression, reduced the Ki-67 positive rate, and increased the infiltration of Grazyme B-positive cells in osteosarcoma tumors (Fig. 8C–E).

## 4 Discussion

Osteosarcoma is a common malignancy with a high degree of malignancy and poor prognosis,^30^^,^^31^ accounting for 44.6 % of bone tumors.^32^ It is most common in children and adolescents.^33^^,^^34^ Despite advancements in medical technology, the 5-year survival rate ramains approximately 20 %, reflecting limited progress in systemic therapies over decades, primarily due to the complexity of its mechanisms of occurrence and metastasis.^3^^,^^4^ Chemotherapy and surgery remain the primary treatment modalities for osteosarcoma, with methotrexate, cisplatin, and doxorubicin as first-line chemotherapy drugs; however, their effectiveness is limited.^33^ The stagnant survival rate over the past 30 years underscores the urgent need to explore the underlying mechanisms of osteosarcoma and develop novel targeted therapies. In our study, we demonstrated that geniposide effectively inhibits the growth of osteosarcoma. To investigate the underlying mechanisms, we performed proteomic analysis, a hypothesis-free approach to identify global protein changes induced by geniposide. KEGG pathway and GO analyses revealed that oxidative phosphorylation plays a crucial role in osteosarcoma growth, suggesting metabolic reprogramming as a central mechanism. Additionally, geniposide promoted Ndufs8 transcription by upregulating OVOL3 expression, thereby suppressing the growth of osteosarcoma.

The uncontrolled growth of tumors requires a significantly higher energy supply compared to normal cells. In order to meet the high demands, tumor cells undergo metabolic reprogramming, shifting from oxidative phosphorylation to anaerobic glycolysis.^35^ This metabolic shift supports rapid cell proliferation by producing ATP inefficiently and accumulating lactate.^36^ Tumor tissues, including osteosarcoma, have significantly higher energy demands compared to normal tissues.^37^^,^^38^ The energy produced by normal mitochondrial oxidative phosphorylation is insufficient to meet these demands, leading to the inhibition of oxidative phosphorylation pathways, a paradoxical adaptation that prioritizes glycolysis despite mitochondrial dysfunction. As a result, osteosarcoma shifts its energy source from oxidative phosphorylation to anaerobic glycolysis. This shift produces more lactate and generates ATP at a faster rate, albeit in an inefficient manner, to support the continuous proliferation of cancer cells (Fig. 4).

The impact of natural compounds on energy metabolism in tumor cells has been studied extensively and is a crucial area in cancer research.^39^ For instance, compounds such as quercetin,^40^ paclitaxel,^41^ ginsenoside,^42^ and have demonstrated promising anti-cancer effects. In our study, we explored the effect of geniposide on osteosarcoma. Based on preliminary dose - response experiments aimed at ensuring efficacy while avoiding systemic toxicity, we selected varying concentrations of geniposide to treat osteosarcoma *in vivo*. After the treatment, we observed a significant reduction in tumor size, which strongly suggests that geniposide can effectively inhibit the growth of osteosarcoma (Fig. 1). To further validate these *in vivo* findings, we conducted *in vitro* experiments. The results showed that geniposide led to a decrease in the colony formation of osteosarcoma cells, which is a reliable indicator of their long - term proliferative capacity. Additionally, CCK-8 assays demonstrated a reduction in cell viability, and there was evidence of promoted apoptosis (Fig. 3D–F). These findings collectively support the inhibitory effect of geniposide on osteosarcoma both *in vivo* and *in vitro*. In order to explore the underlying mechanisms, KEGG pathway analysis suggested that geniposide altered the metabolic process in osteosarcoma, particularly by affecting oxidative phosphorylation (Fig. 2D). Geniposide was found to activate oxidative phosphorylation, a process that is typically suppressed in cancer cells (Fig. 3, Fig. 4). By modulating the disrupted energy supply, geniposide curbs the uncontrolled proliferation of osteosarcoma cells and restores metabolic homeostasis, thereby impeding cancer progression.

The process of oxidative phosphorylation is complex and involves a series of enzymes that convert glucose into energy and metabolites.^43^ Proteomics analysis pinpointed Ndufs8 as a pivotal protein implicated in the activation of oxidative phosphorylation. As an integral component of mitochondrial Complex I, Ndufs8 is indispensable for the proper functioning of the electron transport chain. Previous studies have shown that Ndufs8 is embedded in the inner mitochondrial membrane and plays a critical role in the respiratory electron transport chain .^44^ Ndufs8 maintains oxidative phosphorylation and provides energy for cell growth its loss is associated with mitochondrial disorders and tumor progression. Geniposide increased Ndufs8 levels in osteosarcoma cells, thereby activating oxidative phosphorylation (Fig. 4, Fig. 5A–D). However, few studies have investigated the role of Ndufs8 in cancer, particularly in osteosarcoma, highlighting the novelty of this finding. Intracellular protein levels are regulated by both transcription and degradation processes. In this study, we investigated the transcription regulation of Ndusf8. ChIP-seq analysis suggested that OVOL3 binds to the promoter region of Ndufs8 (Fig. 6A). OVOL3 is a highly conserved gene encoding C2H2 zinc finger transcription factors in mammals.^45^ As a transcription factor, OVOL3 regulates gene expression during various differentiation processes.^19^

We confirmed through ChIP - qPCR and deletion/mutation analysis of the promoter (Fig. 6) that OVOL3 binds to the promoter region of Ndufs8. Our results also showed that OVOL3 enhances the expression of Ndufs8 when the cells are exposed to geniposide. (Fig. 7B). These findings suggest that OVOL3 may play a critical role in activating Ndufs8 transcription. However, we have not yet conducted in-depth studies on the degradation pathways of Ndufs8. We would comprehensively explore the degradation pathways of Ndufs8 (e.g., ubiquitin-proteasome system or autophagy-mediated turnover). Future studies will aim to comprehensively investigate the regulatory mechanisms of geniposide on Ndufs8, including its degradation pathways.

Geniposide upregulates AMPK expression and the AMPK pathway is known to regulate PD-L1 expression.^46^ Therefore, we set out to examine the expression level of PD-L1 in osteosarcoma specimens treated with geniposide. Intriguingly, through flow cytometry and IHC, we found that geniposide significantly downregulated PD-L1 expression in osteosarcoma cells. Moreover, in a syngeneic mouse model, the combination of geniposide and PD-1 inhibitors led to an improved anti - tumor response. IHC analysis further revealed an enhanced T - cell immune response within osteosarcoma tumors when treated with both geniposide and PD-1 inhibitors. These findings strongly suggest a synergistic effect between metabolic reprogramming induced by geniposide and immune checkpoint blockade mediated by PD-1 inhibitors.

Taken together, we evaluated the effects of different concentrations of geniposide on the growth of osteosarcoma. Our results demonstrated that geniposide inhibits the growth of osteosarcoma. Proteomic analysis revealed that oxidative phosphorylation is a key pathway through which geniposide exerts its inhibitory effects, bridging natural compound pharmacology with cancer metabolism. Furthermore, Ndufs8 activation of oxidative phosphorylation was found to reverse osteosarcoma growth, providing a mechanistic target for future therapies. Geniposide enhanced Ndufs8 transcription through upregulation of OVOL3 expression in osteosarcoma. At last, geniposide reduced PD-L1 expression in osteosarcoma cells and enhanced the effectiveness of PD-L1 blockade in vivo. These findings suggest that geniposide is a potential therapeutic agent for osteosarcoma and could be used in combination with PD-L1 blockade to enhance immunotherapy outcomes a strategy warranting clinical validation.

## Data availability

The datasets used and analyzed during the current study are available from the corresponding author on reasonable request.

## Funding

This work was supported by Foshan Military Civilian Integration and Sustainable Development Science and Technology Project (2018AG100091).

## Declaration of competing interest

The authors declare the following financial interests/personal relationships which may be considered as potential competing interestsFanghao Zheng reports was provided by Science and Technology Postgraduate Education and Research Development Office, Office of the Higher Education Commission. If there are other authors, they declare that they have no known competing financial interests or personal relationships that could have appeared to influence the work reported in this paper.
