# Supplementary material for: Geniposide activates the transcription of Ndufs8 and enhances PD-L1 blockade for inhibiting growth of osteosarcoma
Source: J Tradit Complement Med. 2025 Apr 5;16(4):433–45. doi: 10.1016/j.jtcme.2025.04.002 (PMC13316530; doi:10.1016/j.jtcme.2025.04.002)
Supplement: Multimedia component 1 [file mmc1.docx]

Supplemental Table 1 The primers used for RT-qPCR

| Gene | Species | Primer sequence |
| --- | --- | --- |
| Ndufs8 | Human | F:5’- GCCACCATCAACTACCCGTT-3’ |
|  |  | R:5’- CCGCAGTAGATGCACTTGGT-3’ |
| Ndufs8 | Mouse | F:5’- AGCCTGCCACCATCAACTAC-3’ |
|  |  | R:5’- CCTCAATGGTGATGGCCTGT-3’ |
| OVOL3 | Human | F:5’-CTGGTCAGGAGTCGGCGT-3’ |
|  |  | R:5’-CTGCAGTCTGGGATATAGGCATC-3’ |
| OVOL3 | Mouse | F:5’-TCTTTCTTGTGAGGAGTCGGC-3’ |
|  |  | R:5’-TGCAGTCTGGGACATAAGCA-3’ |
| GAPDH | Human | F:5’-GGAGCGAGATCCCTCCAAAAT-3’ |
|  |  | R:5’-GGCTGTTGTCATACTTCTCATGG-3’ |
| GAPDH | Mouse | F:5’-AGGTCGGTGTGAACGGATTTG-3’ |
|  |  | R:5’-GGGGTCGTTGATGGCAACA-3’ |
